# Can the New Humanized Mouse Model Give HIV Research a Boost?

**DOI:** 10.1371/journal.pmed.0050013

**Published:** 2008-01-15

**Authors:** Barbara L Shacklett

## Abstract

The "BLT mouse" holds promise for the preclinical evaluation of microbicides and antiretroviral prophylaxis regimens.

Over the last few months, the medical community has received both good and bad news concerning the AIDS epidemic. The “good” news: the number of individuals infected with HIV worldwide was revised downward and was estimated at 33 million rather than 40 million [1]; although lower than expected, this still represents a staggeringly high figure. The bad news: two highly publicized vaccine trials were prematurely terminated due to a high frequency of seroconversions among vaccine recipients [2]. The obvious message from both of these reports is that more research is needed to uncover better strategies for preventing HIV transmission and more effective treatments for those already infected.

## Limitations of Current Animal Models for HIV

Infection of rhesus macaques with simian immunodeficiency virus (SIV) has provided an excellent nonhuman primate model for studying HIV pathogenesis (reviewed in [3]). SIV is closely related to HIV on a molecular level, its replication may be inhibited by many of the same antiretroviral compounds, and it induces an acquired immunodeficiency syndrome (AIDS) that mimics human AIDS in many important respects. SIV can also be transmitted experimentally to rhesus macaques across the cervicovaginal or rectal mucosa, providing a means of testing microbicides as well as studying the earliest events involved in mucosal transmission. However, the SIV model also has two major disadvantages. First, rhesus macaques are costly and in high demand, and must be housed in accredited primate facilities; there are a limited number of such facilities nationwide. Second, despite its similarity to HIV, SIV and HIV differ in numerous subtle ways, including genetic organization (for example, the *Vpx* gene is unique to SIV; *Vpu* is unique to HIV) and disease course (simian AIDS generally develops within six to 12 months of infection with SIV, while it can take many years for human AIDS to develop after infection with HIV).

**Linked Research Article**

This Perspective discusses the following new study published in *PLoS Medicine:*

Denton PW, Estes JD, Sun Z, Othieno FA, Wei BL, et al. (2008) Antiretroviral pre-exposure prophylaxis prevents vaginal transmission of HIV-1 in humanized BLT mice. PLoS Med 5(1): e16. doi:10.1371/journal.pmed.0050016

J. Victor Garcia and colleagues show that mice with immune systems reconstituted from human bone marrow, liver, and thymus transplants provide a model for prevention of intravaginal HIV infection.

## Why a Mouse Model?

Rodent models for HIV infection are appealing for a number of reasons. Rodents are inexpensive, reproduce quickly, and may be housed in large numbers in a fairly small facility. Experiments may be set up with a large number of replicates. However, several barriers have historically prevented the establishment of a satisfactory rodent model for HIV infection. First, and most importantly, although mice and rats are hosts for a number of retroviruses that are distantly related to HIV, rodent cells are nonpermissive for HIV infection. Attempts to engineer rodents that are permissive for HIV replication and dissemination have been only partially successful [4]. Rats stably expressing human CD4 and CCR5, the major receptor and coreceptor for HIV-1, respectively, are partially permissive [5]; addition of the *human cyclin T1* gene further enhances HIV gene expression, but additional barriers remain [6].

Severe combined immune deficiency or “SCID” mice have served as effective research tools for many years [7]; these mice may either be engrafted with human peripheral blood mononuclear cells (hu-PBL-SCID) [8], or may receive surgically implanted xenografts containing human fetal thymus and liver tissue (SCID-hu thy/liv) [7]. The SCID-hu thy/liv model is extensively used for preclinical testing of antiretroviral drugs [9]. However, potential limitations of this model include a limited repertoire of human cell types and limited distribution of the human cells in tissues outside the implants.

Recently, several groups have reported humanized rodent models that appear to at least partially address these issues. In the bone marrow/liver/thymus, or “BLT” mouse, nonobese diabetic (NOD)/SCID mice (which lack endogenous T and B cells) are surgically implanted with fetal thymic and liver organoids, as in the SCID-hu system [10]. The mice are then sublethally irradiated and transplanted with autologous CD34^+^ stem cells obtained from fetal liver; these cells then take up residence in the murine bone marrow. Thus, the mice undergo a bone marrow transplant, receiving human stem cells that are autologous to their human thy/liv implants. Mice prepared in this way show an impressive range of human cells in peripheral blood, including mature T and B lymphocytes, monocytes, macrophages, and dendritic cells. Equally importantly, they show extensive infiltration of organs and tissues with human cells, including liver, lung, and gastrointestinal tract [10,11].

## A New Study of BLT Mice

In a new study published in *PLoS Medicine,* Paul Denton and colleagues have extended these findings to show that the female reproductive tissues of BLT mice are adequately reconstituted with HIV-susceptible human CD4^+^ T cells, as well as other relevant populations [12]. Atraumatic intravaginal exposure of these mice to HIV-1 led to systemic HIV-1 infection coupled with a rapid loss of human CD4^+^ T cells from the gastrointestinal mucosa, now known to be a hallmark of acute HIV infection in humans and of SIV infection in macaques. In their study, vaginal HIV infection could be prevented by pre-exposure prophylaxis using a combination of emtricitabine and tenofovir disoproxil fumarate. Thus, this proof-of-concept study shows that the BLT mouse holds promise for the preclinical evaluation of microbicides and antiretroviral prophylaxis regimens.

Nevertheless, it would be premature to state that these findings have clear predictive value for clinical medicine. Importantly, it remains unknown whether pre-exposure prophylaxis can indeed protect humans from HIV infection [13]. Thus, the fact that such a regimen can protect BLT mice is encouraging but does not in itself constitute a validation of such prophylaxis in humans. Differences between mice and humans that will require additional attention in validating the BLT model include: (1) differences in dosing of antiretroviral drugs and viral inoculum; (2) the presence of coinfections; (3) atraumatic versus traumatic exposure; and (4) basic differences in the anatomy and physiology of the female reproductive tract.

## Conclusions

From the perspective of a basic scientist searching for improved animal models in which to study human disease, the present study represents a clear advance in the field. However, given the uncertainties that remain, the BLT mouse is unlikely to replace the SIV model for studying pathogenesis and transmission. At this stage, the most prudent approach is to consider the new humanized rodents and the more established, nonhuman primate models as complementary systems, both of which can yield useful information but neither of which is infallible.

## Abbreviations

BLT: bone marrow/liver/thymus
SCID: severe combined immune deficiency
SIV: simian immunodeficiency virus
